# Genomic Characterization of a *Tomato Yellow Mottle-Associated Virus* Collected from a Field Tomato Plant in Chengdu, Southwestern China

**DOI:** 10.1128/mra.00297-22

**Published:** 2022-05-23

**Authors:** Meifang Peng, Jingjing Huang, Tao Lang, Xiaomin Lin, Xiaoli Fan, Kegui Chen

**Affiliations:** a Institute of Biotechnology and Nuclear Technology, Sichuan Academy of Agricultural Sciences, Chengdu, Sichuan, China; b Sichuan Academy of Agricultural Sciences, Chengdu, Sichuan, China; Queens College CUNY

## Abstract

Here, we report the genomic sequence and genetic variations of a *Tomato yellow mottle-associated virus*. The virus isolated from a field tomato (Solanum lycopersicum) plant in Chengdu, southwestern China, was sequenced via both Illumina and Sanger technologies. Phylogeny indicates that its genome is close to the reported virus sequence from *S. lycopersicum* collected in 2013 but far from Solanum nigrum collected in 2020.

## ANNOUNCEMENT

**FIG 1:**
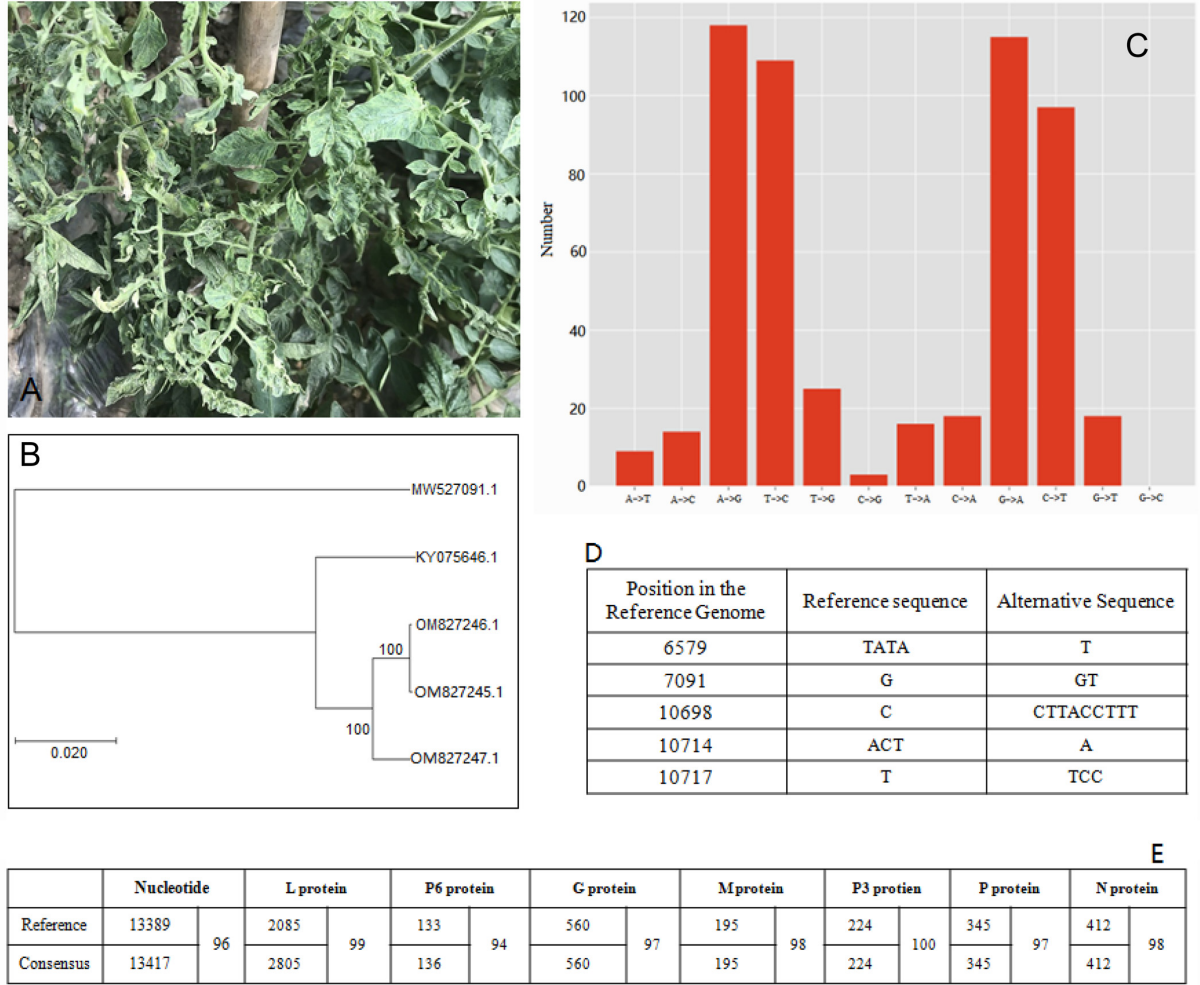
(A) TYMaV-infected tomato plant. (B) Phylogenic tree of the five virus genome sequences available in GenBank. (C) Statistics of nucleotide mutations identified in the virus genome by UMI RNA-seq. (D) Indels identified in the virus genome by UMI RNA-seq. (E) Blast results between the virus consensus sequence and the virus reference genome. The nucleotide column is BLASTN of the genomic nucleotide sequences and all the rest columns are Blantp of the amino acid sequences of 7 ORFs. The two numbers in the first sub columns of each column are sequence length of nucleotides or amino acids receptively for the reference and consensus and the following number in next sub column is identity percentage between the two sequences.

*Tomato yellow mottle-associated virus* (TYMaV) is a newly emerged *Cytorhabdovirus* (family *Betarhabdovirinae*) causing serious damage in tomato fields (1). The virus sample was collected in the summer of 2020 from leaves of an infected tomato plant (Fig. 1A) in Chengdu (104°07′E, 30°37′N), China. Total RNA in this study was obtained by TRIzol (Invitrogen) extraction. A small RNA library was prepared using TruSeq small RNA library preparation kits (Illumina). Sequencing was carried out on a HiSeq 2500 (Illumina) instrument. In total, 15,937,340 raw reads obtained were trimmed and filtered with the next-generation sequencing (NGS) quality-control (QC) Toolkit v2.3.3, resulting in 14,332,450 clean reads. Bowtie v1.0.0 (2) was used to remove the reads of rRNA, tRNA, small nuclear RNA, small nucleolar RNA, and repeat sequences from the clean reads. The obtained 9,716,884 reads were *de novo* assembled using Velvet v1.0 (3), resulting in 531 contigs mapped to the reference genome (GenBank, NC_034240) with 84.25% coverage viewed on IGV 2.8.12 (4).

The second Illumina sequencing was conducted via unique molecular identifier (UMI) transcriptome sequencing (RNA-seq) (5). The library was generated with the RNA using a SeqHealth mRNA-seq library prep kit (Illumina). Trimmomatic v0.39 (6) was applied to remove reads containing adaptors, and reads with low quality from the raw reads of 88,845,324 were obtained via an Illumina HiSeq X Ten sequencer. The identified reads of 84,651,220 were analyzed using the kcUID software suite (https://github.com/KC-UID/KC-UID), resulting in 70,595,106 reads called unique identifier reads. Finally, 36 contigs from the unique identifier reads were assigned via Velvet v1.0 to the virus reference genome. The assembly genomic consensus (GenBank, OM827245), viewed on IGV 2.8.12, contains 13,417 nucleotides (nt) with an extra 28 nt in front of 5′ terminus of the reference genome. An analysis using GATK 4.1.7.0 (https://github.com/broadinstitute/gatk/releases) revealed 542 single nucleotide polymorphisms (SNPs) with majority in transitions (Fig. 1C) and 5 indels (Fig. 1D) in the genome. Seven open reading frames (ORFs) in the antigenomic RNA strand were identified with at least 94% identities of amino acids to the respective reference homologues (Fig. 1E).

Sanger sequencing of the PCR-amplified fragments with the primers listed in Table 1 was carried out to confirm the virus genome sequence. The template was synthesized with the PrimeScript reverse transcriptase (RT) reagent kit with the genomic DNA (gDNA) Eraser (Takara Bio). The PCR fragments were further cloned into a pMD 19-T vector cloning kit (Takara Bio) and sequenced. The obtained sequences were assembled with termini from the first Illumina sequence using BioEdit v7.2.6.1. Two assembly molecules of the genome (GenBank, OM827246 and OM827247) were identified with 98% identities of nucleotide sequences. BLASTN with the reference genome revealed that a T insertion in position 7117 happened to both, which is consistent with the observed inserted T polymorphism at the position 7091 in the reference genome (Fig. 1D). Phylogeny using MEGA11 in ClustalW alignment and neighbor-joining tree construct (7) demonstrated a tight cluster of the three sequences in this report separating from *S. lycopersicum* and further from *S. nigrum* (Fig. 1B). All software used in this study was run at default settings.

**TABLE 1:** Primers for PCR amplification in this study

Fragment	Primer	Sequence (5′–3′)
01	FQRV-01F	TCAGTGGTTCCGTCATTATGTAGTA
	FQRV-01R	GATCTAGAGAAGGCCACTCGATG
02	FQRV-02F	CATCGAGTGGCCTTCTCTAGATC
	FQRV-02R	GATGGTGAGAGGCTTCTCTGATC
03	FQRV-03F	GATCAGAGAAGCCTCTCACCATC
	FQRV-03R	CAGAACCTCGGCGTCTATAGG
04	FQRV-04F	CAGAACCTCGGCGTCTATAGG
	FQRV-04R	TGCATGAAGCCCGATCAGAAT
05	FQRV-05F	GACACCTCCTCGTTTTAACTCTATTG
	FQRV-05R	GACTGCTCATCGCTGTGAAAGA
06	FQRV-06F	TCTTTCACAGCGATGAGCAGTC
	FQRV-06R	CAGCGGATCAATGAGGCAT
07	FQRV-07F	ATGCCTCATTGATCCGCTG
	FQRV-07R	CATTGCAATTGTCGAACACTGAC

### Data availability.

The two Illumina sequencing raw data sets were submitted to the NCBI SRA database with SRX14182798 (small RNA-seq) and SRX14182870 (UMI RNA-seq). The assembly consensus sequences were deposited in GenBank under OM827245, and other two molecules were deposited there under OM827246 and OM827247.
